# MPGN and mixed cryoglobulinemia in a patient with hepatitis C – new treatment implications and renal outcomes 

**DOI:** 10.5414/CNCS109099

**Published:** 2017-10-23

**Authors:** Shannon B. Palombo, Eric C. Wendel, Laura R. Kidd, Farshid Yazdi, Mihran V. Naljayan

**Affiliations:** 1 Internal Medicine/Pediatrics,; 2 School of Medicine, LSUHSC-NO,; 3 Department of Pathology & Laboratory Medicine, Tulane University, and; 4 School of Medicine, Section of Nephrology and Hypertension, LSUHSC-NO, New Orleans, LA, USA

**Keywords:** cryoglobulinemia, membranoproliferative glomerulonephritis (MPGN), hepatitis C (HCV), direct-acting antivirals (DAA)

## Abstract

Abstract. Introduction: The association of hepatitis C virus (HCV), cryoglobulinemia, and membranoproliferative glomerulonephritis (MPGN) is well known. Treatment of underlying HCV infection has greatly improved in recent years with the introduction of direct-acting antivirals (DAA), which have demonstrated curative sustained viral response (SVR) rates for select viral genotypes with the added benefit of less drug side effects. However, a mainstay of newer DAAs is sofosbuvir, which is contraindicated in patients with severe renal impairment. Case history: We are reporting the case of a 65-year-old female with chronic systolic heart failure, hypertension, and chronic HCV genotype 1b with biopsy-proven type I MPGN with cryoglobulinemia type II, who presented with rapidly progressive renal failure requiring emergent hemodialysis. After initiation of DAA therapy including ombitasvir-paritaprevir-ritonavir plus dasabuvir, in conjunction with plasmapheresis, corticosteroids, and rituximab, there was significant improvement in renal function such that hemodialysis was no longer needed. Discussion: This patient’s HCV treatment is estimated to induce a greater than 90% SVR, which is notably promising for the reduction and/or reversal of HCV-related glomerulopathy. Most recent HCV guidelines from 2015 recommend this regimen; however, there is little data to evaluate the safety and efficacy of treatment. Therefore, it is valuable to report positive preliminary results at this time. Overall, we anticipate this treatment regimen to become a basis in the management of HCV-related renal disease; however, larger studies will still be needed to prove its efficacy in improving renal outcomes.

## Introduction 

The association of hepatitis C virus (HCV), cryoglobulinemia, and membranoproliferative glomerulonephritis (MPGN) is well known. 90% of patients with mixed cryoglobulinemia are HCV-positive; 50% of patients with chronic hepatitis C have circulating cryoglobulins; and 80% of patients with cryoglobulinemia develop MPGN [1]. 

WHO estimates HCV prevalence accounts for 3% of the world’s population, which estimates to 170 million people [2], 2.7 – 3.9 million of which are in the United States. Recent studies report that 2% of patients with hepatitis C have a glomerular filtration rate (GFR) < 30%, of which 0.9% are associated with cryoglobulinemia [3]. Cryoglobulinemia is characterized by palpable purpura, arthralgias, fever, and renal disease including hematuria, proteinuria, and renal impairment. Type II is more common in the 4^th^ – 5^th^ decade of life, occurring more frequently in women [1]. 

Treatment of hepatitis C-associated cryoglobulinemia with MPGN is a three-part strategy in patients with rapidly progressive glomerulonephritis or nephrotic syndrome. Part one and two consist of B-cell depletion to reduce the production of cryoglobulins and non-specific immunosuppression to target vasculitic inflammation. Part three is antiviral therapy with the goal of treating the underlying infection to inhibit immune complex formation and resulting vasculitis [4, 5]. 

Previous treatment of HCV included ribavirin and pegylated interferon (PEG-IFN), both associated with severe side effects. However, treatment of HCV infection has greatly improved in recent years with the introduction of direct-acting antivirals (DAA), which have demonstrated curative sustained viral response (SVR) rates for select viral genotypes with the added benefit of less drug side effects. However, a mainstay of newer DAAs is sofosbuvir, which is contraindicated in patients with severe renal impairment [5]. The safety and efficacy of new HCV guidelines for treatment of patients with renal failure is still an ongoing investigation at that time. We present a case of hepatitis C-induced cryoglobulinemia, which precipitated acute renal failure and led to emergent treatment based on these new HCV treatment regimens. We believe this case is important as it is the first reported case of HCV-related cryoglobulinemia with MPGN that led to renal failure requiring dialysis with successful treatment using DAAs. 

## Case description 

**Figure 1.**
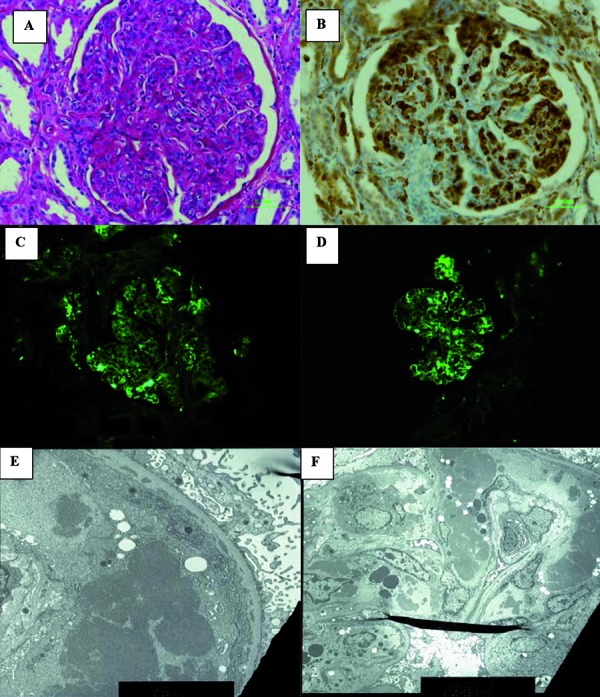
A: light microscopy (PAS); B: Tri-chrome; C: IgG deposits; D: κ-deposits; E, F: Subendothelial electron-dense deposits consistent with cryoglobulin immune complexes.

Our patient is a 65-year-old African-American female who presented with shortness of breath and bilateral lower-extremity edema for 2 weeks. Her past medical history was significant for hypertension, coronary artery disease, and chronic systolic heart failure (ejection fraction 35%). She reported a 50-pack-year tobacco history in addition to remote history of intravenous drug use. On physical exam, the patient was noted to have bibasilar crackles and bilateral lower extremity pitting edema. Brain natriuretic peptide (BNP)****was elevated at 954 pg/mL with significant pulmonary edema noted on chest X-ray. The patient was admitted for treatment of acute on chronic systolic heart failure. Additional admission labs showed acute kidney injury (AKI) with creatinine of 1.39 mg/dL, with unknown baseline for comparison. Urinalysis was significant for granular casts, dysmorphic red blood cells, and proteinuria of > 500 mg/dL with a protein/creatinine ratio of 13,216 mg/g. A hepatitis panel revealed positive antibodies to HCV, and PCR showed 1b genotype and a viral load of 4,975,007. C4 complement levels were decreased (< 8 mg/dL; reference range 18 – 55 mg/dL), and rheumatoid factor level was positive (45.8 IU/mL, reference range < 20.0 IU/mL). These laboratory findings were consistent with cryoglobulinemia with cryocrit of 3%. On hospital day 14, a percutaneous kidney biopsy revealed membranoproliferative glomerulonephritis type I with mixed cryoglobulinemia type II with minimal fibrosis and underlying acute tubular necrosis (Figure 1). On light microscopy, there were focal glomeruli with segmental hyaline thrombi noted. One glomerulus had a possible fibrous crescent, but no cellular crescents or segmental necrosis was seen. By immunofluorescence, there is granular capillary and mesangial staining and segmental intracapillary hyaline thrombi staining with IgG (2+), IgM (3+), κ (3+) and λ (1+) light chains. Electron microscopy shows findings consistent with cryoglobulinemia. 

By hospital day 16, the patient’s kidney function continued to worsen with a peak creatinine of 6.08 mg/dL in addition to the development of uremic symptoms. Hemodialysis was initiated for 3 days in total. On hospital day 19, the patient began three-part treatment for hepatitis C with associated cryoglobulinemia beginning with plasmapheresis for 5 days and a 12-week regimen of antiviral therapy with ombitasvir, paritaprevir-ritonavir plus dasabuvir. Immunosuppression with solumedrol and rituximab infusions was initiated on day 24. The patient was discharged on hospital day 33 with a creatinine of 2.35 mg/dL and no additional sessions of hemodialysis. On follow-up day 57, her hepatitis C viral load was undetectable. Shortly thereafter, her creatinine was 1.08 mg/dL with an improved protein/creatinine ratio of 677 mg/g and has subsequently remained stable on routine outpatient monitoring. 

## Discussion 

The 2015 HCV guidelines recommend new treatment strategies for HCV cryoglobulinemia; however, there is little data to evaluate the safety and efficacy in patients with severe kidney disease. A case report from 2014 describes DAA telaprevir as a successful therapy for the reversal of renal failure in a patient with HCV cryoglobulinemia; however, this was used in conjunction with PEG-IFN and ribavirin [7] with new recommendations now avoiding these combination therapies. The AASLD-ISDA 2015 guidelines state that for patients infected with hepatitis C genotype 1b, treatment strategies include ledipasvir/sofosbuvir, ritonavir-boosted paritaprevir, ombitasvir, and dasabuvir, or sofosbuvir with simeprevir with or without ribavirin. These are all treatment strategies for patients with normal kidney function. They also recommend that if estimated glomerular filtration rate (eGFR) > 30 mL/min/1.73m^2^, then no dose adjustment is required to the above treatment regimens. In patients with eGFR < 30 mL/min/1.73m^2^, combination therapy of ritonavir-boosted paritaprevir, dasabuvir, and ombitasvir, with or without ribavirin can be used [6]. This treatment is only for use in genotype 1a and 1b HCV and should not be used in patients with eGFR < 15 mL/min/1.73m^2^. Currently, there is an ongoing trial (RUBY-1) evaluating patients with severe renal disease that appears to show safety and efficacy in patients using ritonavir-boosted paritaprevir, dasabuvir, and ombitasvir with ribavirin for patients with hepatitis C genotype 1 on hemodialysis. The results of this trial are still pending. Roth et al. [5] published a phase 3 trial of grazoprevir and elbasvir in patients with CKD stage 4 – 5 or on hemodialysis (C-Surfer Study) which showed sustained virological response in 99% of patients after 12 weeks. This study showed a low rate of adverse events in these patients [5]. Current recommendations from the AASLD/IDSA are against using PEG-IFN and ribavirin for hepatitis C with severe renal disease (eGFR < 15 mL/min/1.73m^2^) until renal function improves [6]. 

Our case is valuable to report positive results for marked improvement and stabilization of renal function for a patient with HCV-induced cryoglobulinemia with associated renal failure using newer treatment options available for hepatitis C. However, larger patient population studies are needed to determine prognostic efficacy, morbidity, and mortality outcomes. 

## Conflict of interest 

There are no financial disclosures, and this study did not require any funding.
